# Banhabaekchulchunma-tang, a traditional herbal formula attenuates absolute ethanol-induced gastric injury by enhancing the antioxidant status

**DOI:** 10.1186/1472-6882-13-170

**Published:** 2013-07-12

**Authors:** In-Sik Shin, Woo-Young Jeon, Hyeun-Kyoo Shin, Sin-Woo Cha, Mee-Young Lee

**Affiliations:** 1 Basic Herbal Medicine Research Group, Korea Institute of Oriental Medicine, 483 Expo-ro, Yusung-gu, Daejeon 305-811, Republic of Korea; 2 Department of Nonclinical Studies, Korea Institute of Toxicology, 141 Gajeong-ro, Yusung-gu, Daejeon 305-343, Republic of Korea

**Keywords:** Banhabaekchulchunma-tang, Herbal formula, Ethanol, Gastric injury, Antioxidant

## Abstract

**Background:**

Banhabaekchulchunma-tang (hange-byakujutsu-tenma-to in Japanese and banxia-baizhu-tianma-tang in Chinese) is a mixture of fourteen herbs. It is used traditionally for the treatment of anemia, anorexia, general weakness, and female infertility in China, Japan, and Korea. In this study, we investigated the protective effects of a Banhabaekchulchunma-tang water extract (BCT) against ethanol-induced acute gastric injury in rats.

**Methods:**

Gastric injury was induced by intragastric administration of 5 mL/kg body weight of absolute ethanol to each rat. The positive control group and the BCT group were given oral doses of omeprazole (50 mg/kg) or BCT (400 mg/kg), respectively, 2 h prior to the administration of absolute ethanol. The stomach of each animal was excised and examined for gastric mucosal lesions. To confirm the protective effects of BCT, we evaluated the degree of lipid peroxidation, the level of reduced glutathione (GSH), and the activities of the antioxidant enzymes catalase, glutathione-S-transferase, glutathione peroxidase, and glutathione reductase in the stomach. In addition, we conducted an acute toxicity study to evaluate the safety of BCT according to OECD guideline.

**Results:**

BCT reduced ethanol-induced hemorrhage, hyperemia, and loss of epithelial cell in the gastric mucosa. BCT reduced the increased lipid peroxidation associated with ethanol-induced acute gastric lesions, and increased the mucosal GSH content and the activities of antioxidant enzymes. In addition, BCT did not cause any adverse effects at up to 5000 mg/kg.

**Conclusions:**

These results indicate that BCT protects the gastric mucosa against ethanol-induced gastric injury by increasing the antioxidant status. We suggest that BCT could be developed as an effective drug for the treatment of gastric injury caused by alcohol intake.

## Background

Oxidative stress is closely related to the pathogenesis of various diseases and it is postulated to play a crucial role in the development of gastric mucosal injuries induced by ethanol in rats [1]. Oxidative stress is induced by reactive oxygen species (ROS) generated by the xanthine-xanthine oxidase system and activated neutrophils, which leads to tissue lipid peroxidation. When combined with gastric secretions this leads to damage and cellular death, which can result in hemorrhage, erosion, ulcers, and loss of gastric mucosa [2]. To protect tissues from the damage induced by oxidative stress, cells contain antioxidant defense systems including catalase, glutathione (GSH), glutathione-S-transferase (GST), glutathione peroxidase (GPx), glutathione reductase (GR) and superoxide dismutase (SOD) [3]. The antioxidant defense system protects against the oxidative stress induced by ethanol intake by scavenging oxygen-derived free radicals directly or increasing levels of radical scavengers, such as sulfhydryl compounds [4]. Several researchers have investigated the effects of many herbal medicines in protection against ethanol-induced gastric mucosal injuries by focusing on increases in the antioxidant defense system. Indeed, previous studies have demonstrated such increases with several herbal medicines, such as Argyreia speciosa, Orthosiphon stamineus, Pithecellobium jiringa, and propolis [5-8].

Banhabaekchulchunma-tang (BCT) is a traditional polyherbal mixture that is used clinically in Asia as a remedy for hypertension and headaches [9]. Thus, several researchers have investigated the vasodilatory effects of BCT [9-11]. However, there have been evaluations of other pharmacological effects of BCT. The composition of BCT is as follows: *Pinelliae Tuber, Citri Unshius Pericarpium, Hordei Fructus Germinatus, Atractylodis Rhizoma Alba, Massa Medicata Fermentata, Poria Sclerotium, Atractylodis Rhizoma, Gastrodiae Rhizoma, Gingen Radix, Astragali Radix, Alismatis Rhizoma, Zingiberis Rhizoma, Phellodendri Cortex, Zingiberis Rhizoma Crudus.* The crude herbs also have anti-inflammatory, antioxidant, and gastroprotective effects [12-19]. Based on the properties of the individual herbs, we hypothesized that BCT may protect the gastric mucosa from oxidative stress induced by ethanol administration by enhancing the antioxidant system. Therefore, we evaluated the protective effect of BCT against ethanol-induced acute gastric injury in rats.

## Methods

### Preparation of BCT

The BCT was prepared in our laboratory (Table 1) from a mixture of chopped crude herbs. Befores performing the study, the identity of each crude herb was confirmed by Professor Je-Hyun Lee of Dongguk University (Cyeongju, Korea). BCT was extracted in distilled water at 100°C for 2 h. The solution was evaporated to dryness and freeze-dried (Extraction yield: 17.6%).

**Table 1:** Composition of BCT

**Latin name**	**Amount (g)**	**Origin**
*Pinelliae tuber*	5.63	China
*Citri unshius pericarpium*	5.63	Korea
*Hordei fructus germinatus*	5.63	Korea
*Atractylodis rhizoma alba*	3.75	China
*Massa medicata fermentata*	3.75	Korea
*Poria sclerotium*	1.88	Korea
*Atractylodis rhizoma*	1.88	China
*Gastrodiae rhizoma*	1.88	Korea
*Ginseng radix*	1.88	Korea
*Astragali radix*	1.88	Korea
*Alismatis rhizoma*	1.88	Korea
*Zingiberis rhizoma*	1.13	Korea
*Phellodendri cortex*	0.75	China
*Zingiberis rhizoma crudus*	6.25	Korea
Total amount	43.75	

### Acute toxicity study

Male and female five week old Sprague–Dawley (SD) rats were purchased from a specific pathogen-free facility at the Orient Bio Co. (Seoul, Korea) and were used after one week of quarantine and acclimatization. All animals were housed in a room maintained at 23 ± 3°C with a relative humidity of 50%, artificial lighting from 08:00 to 20:00 and with 10 to 20 air changes per hour. The animals were fed a commercial pellet diet (PMI Nutrition International, VA, USA) and sterilized tap water *ad libitum* following UV irradiation and filtration. The acute toxicity study was performed in compliance with the test guidelines of the Korea Food and Drug Administration (KFDA) under the Good Laboratory Practice Regulations for Nonclinical Laboratory Studies. The study protocol was approved by the Institutional Animal Care and Use Committee at the Korea Institute of Toxicology (accredited by AALAC International, 1998).

In the preliminary study, a single oral administration of BCT did not induce any toxic effects at a dose level up to 5,000 mg/kg. Based on this results, a dose of 5,000 mg/kg was selected as the limited dose, as recommended by OECD test guidelines. Ten rats from each sex were assigned randomly to two groups with five rats in each group, and the animals received a single 5,000 mg/kg dose by gavage. The vehicle control rats received an equivalent volume of distilled water. After oral administration, all abnormal clinical signs were recorded before and after dosing at least twice a day. The body weight was measured on the day of dosing (Day 1) immediately before treatment, and on Days 2, 4, 8, and 15. On the scheduled termination date (Day 15), all surviving animals were anesthetized using carbon dioxide and sacrificed by exsanguination from the aorta. Complete gross postmortem examinations were performed on all animals.

### Ethanol-induced gastric mucosal injuries

Specific pathogen-free male SD rats that weighed 200–250 g (aged six weeks) were purchased from Deahan Biolink Co., Ltd. (Chungbuk, Korea) and used after one week of quarantine and acclimatization. The animals were kept in a room at 23 ± 3°C with a relative humidity of 50% under a controlled 12 h/12 h light/dark cycle. The rats were given standard rodent chow and sterilized tap water *ad libitum*. All experimental procedures were performed in compliance with the NIH Guidelines for the care and use of laboratory animals and the National Animal Welfare Law of Korea.

Acute gastric lesions were induced by intragastric administration of absolute ethanol, according to a previously described method [20]. Twenty eight rats were allocated to four groups and fasted for 18 h before the experiment. The rats in the control group were given phosphate buffered saline (PBS) orally (5 mL/kg body weight) and the absolute ethanol group (EtOH group) received absolute ethanol (5 mL/kg body weight) by oral gavage. Rats in the positive control group were given omeprazole (50 mg/kg body weight) by oral administration 2 h prior to the administration of absolute ethanol. Omeprazole was used as a positive control drug because it possesses anti-inflammatory and antioxidant activities and has been used widely for the treatment of gastritis. The BCT dose was based on the preliminary study. In the preliminary study, BCT was administered to SD rats at dose levels of 200 and 400 mg/kg. A greater reduction in gastric mucosal lesions was detected in 400 mg/kg treated animals compared with 200 mg/kg treated animals during macroscopic examinations. The fourth group received BCT (400 mg/kg body weight) 2 h prior to absolute ethanol administration.

### Gross gastric mucosa findings

Animals were sacrificed with an overdose of 50 mg/kg pentobarbital, which was administered 1 h after they received the absolute ethanol treatment. The stomach was removed from each animal, and opened along the greater curvature. The tissue was rinsed gently in PBS. The stomach was stretched out on a piece of cork with the mucosal surface facing upward and it was examined in the standard position for gastric mucosal lesions. Photographs of hemorrhagic erosions in the stomach were captured using Photometric Quantix digital camera. Quantitative analysis of gastric mucosal injury index was determined the representative photographs using an image analyzer (Molecular Devices, Inc., CA, USA). After the gastric lesions were photographed, the stomach tissue was stored at −70°C before biochemical analysis.

### Biochemical analysis

The stomach tissue was cut into small pieces and homogenized (1/10 w/v) with tissue lysis/extraction reagent and a protease inhibitor (Sigma, MI, USA). The homogenates were centrifuged at 12,000 rpm for 10 min at 4°C to precipitate any cell debris and the supernatant was used to measure the levels of malondialdehyde (MDA), GSH, catalase, GST, GPx, and GR. The total protein was determined using a protein assay reagent (Bio-Rad Laboratories).

Lipid peroxidation was estimated by determining the MDA content using a thiobarbituric acid reactive substances (TBARS) assay kit (BioAssay Systems, CA, USA). In brief, a 100 μL aliquot of homogenate was mixed with 100 μL of 10% trichloroacetic acid and incubated for 15 min on ice. The mixture was centrifuged at 12,000 rpm for 5 min at 4°C and 200 μL of the supernatant was mixed with 200 μL of thiobarbituric acid and incubated at 100°C for 60 min. After the mixture cooled, the absorbance was measured at 535 nm. The results were expressed as nmol of MDA/mg protein. The levels of reduced GSH were measured using a GSH assay kit (Cayman, AnnArbor, MI, USA), which involved an optimized enzymatic recycling method and GR. The sulfhydryl group of GSH reacts with 5,5-ditho-bis-2-nitrobenzoic acid (DTNB) and produces yellow colored 5-thio-2-nitrobenzoic acid (TNB). A mixed disulfide, GSTNB (between GSH and TNB), is also produced that is reduced by GR to recycle GSH, thereby producing more TNB. The rate of TNB production is directly proportional to this recycling reaction which is in turn directly proportional to the concentration of GSH in the sample. The absorbance of TNB at 410 nm was used to estimate the amount of GSH in the sample. The GSH level was expressed as μmol/mg protein.

The GST activity was determined by measuring the conjugation of 1-chloro-2,4-dinitrobenzene (CDNB) with reduced GSH using a GST assay kit (Cayman). Conjugation is accompanied by an increase in the absorbance at 340 nm and the rate of increase is directly proportional to the GST activity in the sample. The GPx activity was measured indirectly via a coupled reaction with GR using a GPx assay kit (Cayman). Oxidized GSH, which is produced after the reduction of hydroperoxide by GPx, is recycled to its reduced state by GR and nicotinamide adenine dinucleotide phosphate (NADPH). The oxidation of NADPH to NADP^+^ is accompanied by a decrease in the absorbance at 340 nm. The GR activity was determined by measuring the rate of NADPH oxidation using a GR assay kit (Cayman). The oxidation of NADPH to NADP^+^ is accompanied by a decrease in the absorbance at 340 nm. The catalase activity was measured based on the peroxidatic function of catalase using a catalase assay kit (Cayman). This method is based on the reaction of the enzyme with methanol in the presence of an optimal concentration of hydrogen peroxide. The formaldehyde produced is measured colorimetrically at 540 nm with 4-amino-3-hydrazino-5-mercapto-1,24-triazole (Purpald) as the chromogen. The activities of catalase, GST, GPx, and GR, were expressed as U/mg protein.

### Histololgy

The glandular surface of the stomach was examined histologically. Tissue samples were preserved in 10% buffered formalin and processed for paraffin block preparation. Sections that measured about 4 μm in thickness were cut and stained with hematoxylin and eosin. The extent of gastric mucosal injury was evaluated using light microscopy by an experienced histologist who was blinded to the treatment regimen. The histopathological changes were assessed to previousely described criteria [21].

### Statistical analysis

The data are expressed as means ± the standard error of the mean (SEM). Statistical significance was determined using analysis of variance (ANOVA). If a test detected a significant difference between groups, the data were analyzed by a multiple comparisons procedure using Dunnett’s test. Statistical analyses were performed using SYSTAT version 10. The levels of significance were set as p < 0.05 and p < 0.01.

## Results

### Acute toxicity study of BCT

**Figure 1:**
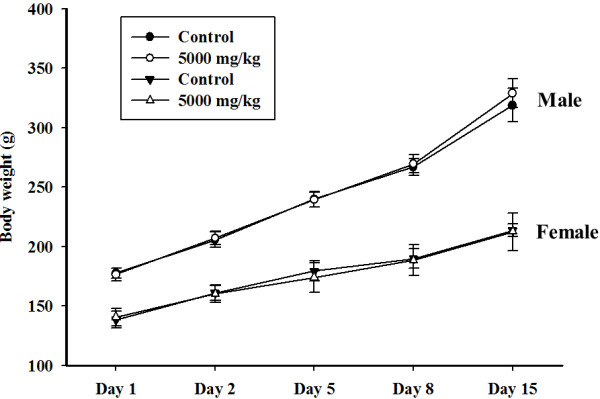
**Body weight changes in animals treated with BCT at dose levels of 0 (●) and 2,000 mg/kg (○) in males and 0 (▼), and 2,000 (Δ) mg/kg in females.** There were no significant differences in the body weights of the BCT treated and control groups.

We conducted an acute toxicity study of BCT to investigate the safety of oral BCT administration. There were no significant differences in the body weight changes of the control and BCT treatment groups for both genders (Figure 1). Throughout the study period, no treatment-related deaths or clinical signs were detected. In addition, BCT treatment did not cause any gross pathological findings in all groups at necropsy.

### Effects of BCT on ethanol-induced acute gastric injury

**Figure 2:**
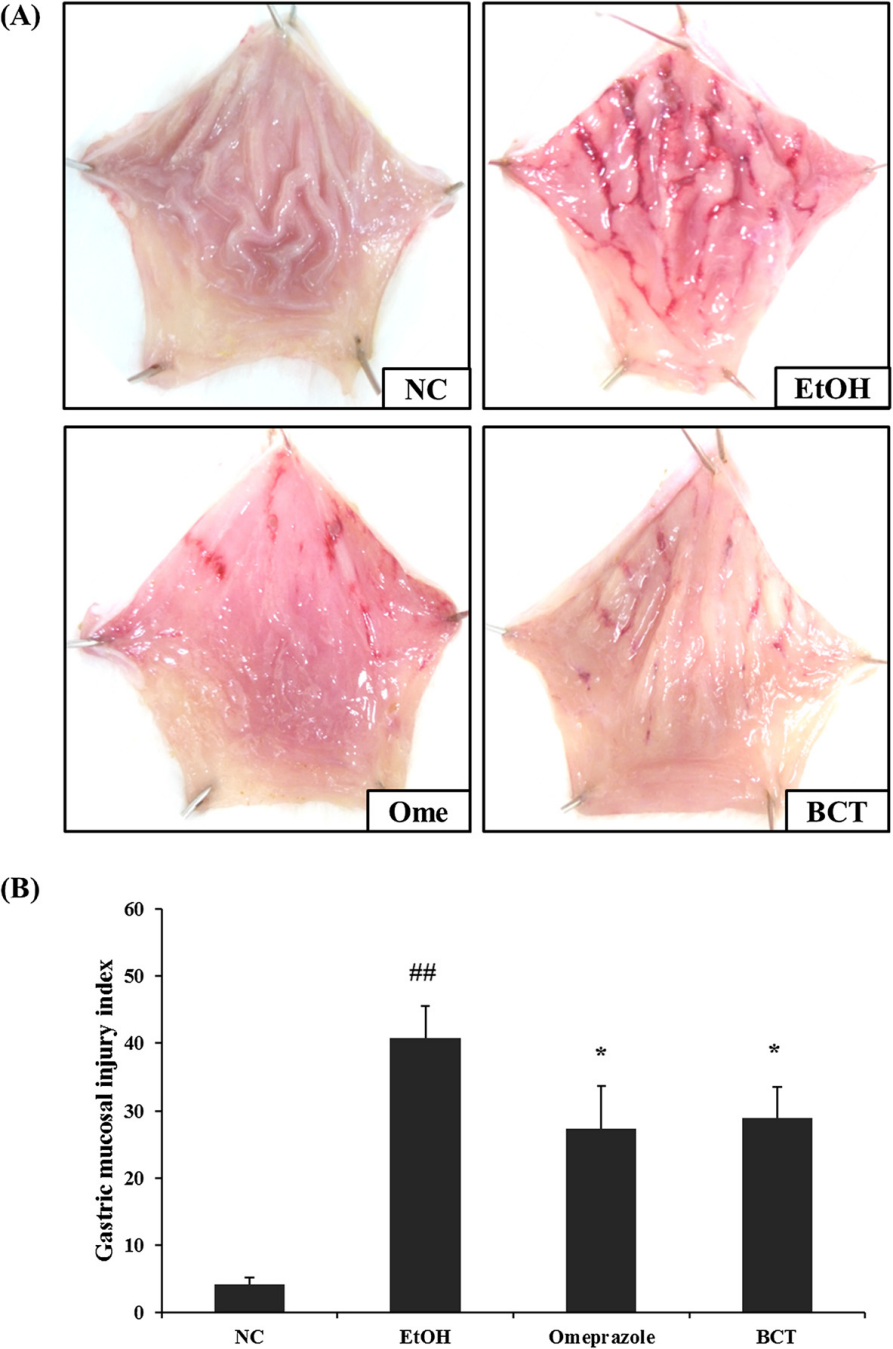
**BCT attenuates gastric mucosa injury induced by absolute ethanol treatment. (A)** Representative photographs of the gastric mucosa, **(B)** quantitative analysis for gastric mucosal injury. Absolute ethanol induced hemorrhages and hyperemia in the gastric mucosa. By contrast, BCT attenuated the gastric mucosal injury induced by absolute ethanol. In quantitative analysis, BCT reduced the elevated gastric injury index induced by absolute ethanol treatment. NC, normal control; EtOH, absolute ethanol treatment group; Ome, absolute ethanol + omeprazole (50 mg/kg); BCT, absolute ethanol + BCT (400 mg/kg). ^##^Significantly different at p < 0.01 compared with the control group, *Significantly different at p < 0.05 compared with the EtOH group.

**Figure 3:**
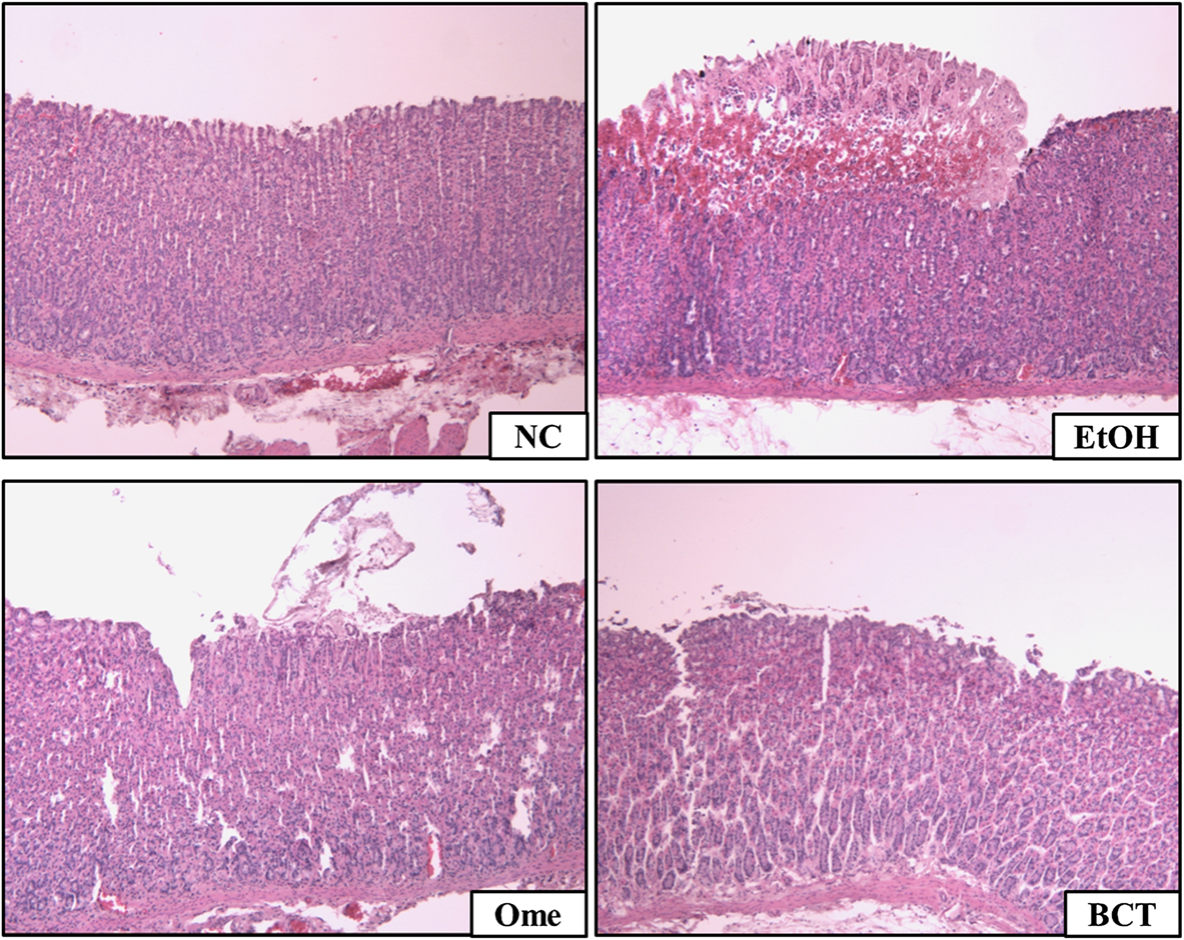
**Histopathology of the gastric mucosa.** Absolute ethanol induced hemorrhagic and the loss of gastric epithelial cells (magnification: ×200). By contrast, BCT attenuated the gastric mucosal injuries induced by absolute ethanol.

Gross examination of the gastric mucosa showed that the EtOH group had gastric mucosal injuries such as hemorrhage and hyperemia (Figure 2A), whereas no abnormalities or lesions were found in the normal control group. In contrast, the omeprazole treated group had attenuated gastric mucosal injuries compared with the EtOH group. The BCT treated group also had attenuated gastric injuries compared with the EtOH group and the omeprazole treated group. In quantitative analysis of gastric mucosal injury index, the BCT treated group also exhibited a significant reduction in gastic mucosal injury index compared with the EtOH group (Figure 2B). In the histopathological examinations, the EtOH group exhibited hemorrhages and the loss of gastric mucosa from the stomach tissue (Figure 3). By contrast, the omeprazole and BCT treated groups experienced reduced acute gastric damage induced by absolute ethanol, such as hemorrhages and loss of the gastric mucosa.

### Effects of BCT on lipid peroxidation and GSH in ethanol induced gastric mucosal injury

**Figure 4:**
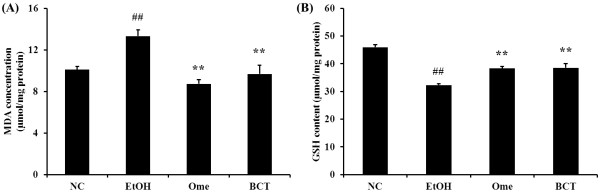
**BCT decreases the gastric MDA concentration and increases the GSH contents in gastric tissue. (A)** MDA concentration, **(B)** GSH contents. Absolute ethanol-induced the elevated MDA and reduced GSH contents in gastric tissue. By contrast , BCT significantly reduced the MDA and increased GSH contents in gastric tissue. Each bar represents the mean ± SEM for six rats. ^##^Significantly different at p < 0.01 compared with the control group, **Significantly different at p < 0.01 compared with the EtOH group.

The concentration of MDA, an end product of lipid peroxidation, was greater in the EtOH group (13.3 ± 0.59 μmol/mg protein, p < 0.01) than the normal controls (10.1 ± 0.66 μmol/mg protein) (Figure 4A). The MDA level in BCT pretreated group significantly lower (9.7 ± 1.89 μmol/mg protein, p < 0.01) compared with the EtOH group. The omeprazole-treated group (8.73 ± 1.08 μmol/mg protein, p < 0.01) also had a significant reduction compared with the EtOH group.

In contrast to the MDA results, the GSH content in the stomachs of the EtOH group (32.3 ± 0.45 μmol/mg protein, p < 0.01) was significantly lower than that in the control group (46.0 ± 0.92 μmol/mg protein), while that of the BCT pretreated group (38.5 ± 1.54 μmol/mg protein, p < 0.01) was higher than that of the EtOH group (Figure 4B).

### Effects of BCT on antioxidant enzymes in ethanol induced gastric mucosal injury

**Figure 5:**
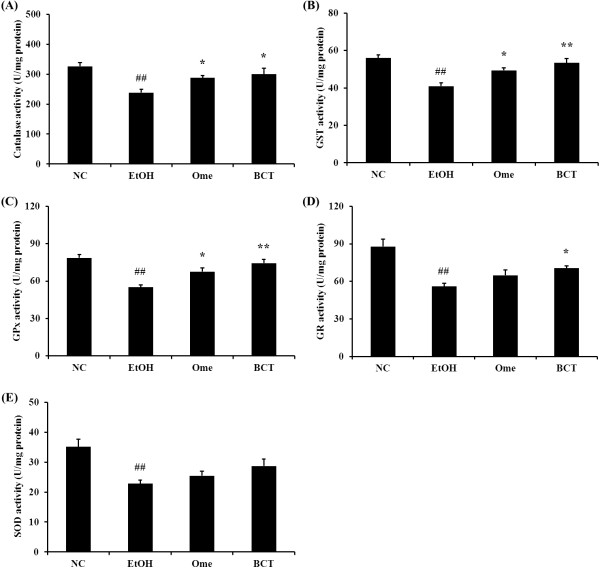
**BCT enhances the activities of antioxidant enzymes in gastric tissue. (A)** catalase, **(B)** GST, **(C)** GPx, **(D)** GR, **(E)** SOD. Absolute ethanol significantly reduced the activities of antioxidant enzymes, including catalase, GST, GPx GR and SOD in gastric tissue. However, administration of BCT markedly increased the activities of antioxidant enzymes. ^##^Significant difference at P < 0.01 compared with the control group, *,**Significantly different at p < 0.05 and < 0.01 compared with the EtOH group, respectively.

As shown in Figure 5A, the catalase activity in the EtOH group (238.1 ± 11.46 U/mg protein, p < 0.01) was lower than that in the control group (326.2 ± 13.00 U/mg protein). However, the BCT treatment produced a significant increase (299.6 ± 19.75 U/mg protein, p < 0.05) in the catalase activity compared with the EtOH group. The omeprazole-treated group (287.9 ± 7.56 U/mg protein, p < 0.05) also exhibited a significantly increased catalase activity compared with the EtOH group. The GST (53.4 ± 2.40 U/mg protein, p < 0.01) and GPx activities (74.4 ± 2.99 U/mg protein, p < 0.01) increased markedly in the BCT-treated group compared with the EtOH group (40.9 ± 1.79 U/mg protein and 55.1 ± 1.97 U/mg protein in GST and GPx, respectively), as well as the catalase activity (Figure 5B and C). In addition, the GR activity in the BCT-treated group (70.5 ± 1.79 U/mg protein, p < 0.05) was higher than that in the EtOH group (56.0 ± 2.55 U/mg protein) (Figure 5D). The SOD activity increased to a greater extent in the BCT-treated group (28.7 ± 2.39 U/mg protein) compared with the EtOH group (22.8 ± 1.20 U/mg protein), but the difference was not significant (Figure 5E).

## Discussion

The gastric mucosa is exposed to various stimuli including ethanol, nonsteroidal anti-inflammatory drugs, bacteria, and viruses. In particular, ethanol intake caused excessive oxidative stress, which induces gastric mucosal damage such as hemorrhage, edema, erosion, ulceration, and loss of epithelial cells. These features were consistent with the gastric lesions induced by absolute ethanol administration in this study. By contrast, the administration of BCT reduced the acute gastric injuries, decreased the MDA levels, and increased the levels of antioxidants including GSH, catalase, GST, GPx, GR, and SOD.

An acute gastric injury model induced by factors that play a role in gastric disorder etiopathogenesis was used to investigate the protective effects of novel materials [22,23]. It is known that stress, alcohol, and steroidal/nonsteroidal inflammatory drugs are some of the factors that increase gastric injury including hemorrhage, erosion, and ulceration [24]. The roles of ROS in the pathogenesis of ethanol induced gastric injury have been demonstrated. ROS cause tissue damage and their levels are reduced by antioxidant defense systems including GSH, catalase, GPx, GR, SOD, and GST [25-27]. These defense systems protect the stomach tissue from the increased ROS production induced by ethanol intake. The gastric injury induced by ethanol intake begins with the formation of lipid radicals in the cell membranes, which damages and destroys the cell membranes [25]. GSH is an endogenous antioxidant component and its activity is related to the thiol group of cysteine in its structure [24]. GSH reacts with peroxides and toxic oxygen radicals such as the hydroxyl ion and singlet oxygen to protect cells from injury [28]. In this study, administration of BCT exhibited a singfincant reduction in the MDA level, a lipid peroxidation product compared with the animals given ethanol (EtOH group). In contrast, the GSH levels were significantly decreased in the EtOH group compared with the normal control, whereas the BCT treated group had higher GSH levels than the EtOH group. These differences are compatible with previous studies [21,27,29].

The levels of enzymatic antioxidant defense components such as catalase, GST, GPx, GR, and SOD were decreased by ethanol intake. By contrast, administration of BCT increased the activities of antioxidant enzymes compared with the EtOH group. SOD is an important antioxidant enzyme that converts superoxide to hydrogen peroxide and oxygen [30]. Thus, it protects aginst the oxidative stress induced in cells and tissues by various stimuli. In addition, catalase catalyzes the decomposition of hydrogen peroxide to water and oxygen, which protects aginst damage to cells and tissues [31]. GST catalyzes the conjugation of GSH via a sulfhydryl group to the electrophilic centers of a wide variety of substrates [24]. These processes detoxify endogenous toxic materials such as peroxidized lipids. GPx is an enzyme with peroxidase activity, which reduces lipid hydroperoxides and free hydrogen peroxide to water [26]. GR is an important antioxidant enzyme that reduces glutathione disulfide to the sulfhydryl form of GSH [32]. Many previous studies have demonstrated that ethanol intake caused reductions in the antioxidant defense system and that several antioxidant materials protect the cells and tissue against toxic materials by enhancing the antioxidant defense system [21,24,29]. These reports agree with the results of the present study except for results of SOD. In this study, oral administration of absolute ethanol notably decreased the activities of antioxidant enzymes, but administration of BCT significantly elevated the activities of enzymes in gastric tissue. Although BCT did not significantly incrased SOD activity; however, the lack of an increase in SOD activity may reflect a lack of substrate for this enzyme [22]. Thus, our findings indicate that BCT administration can protect the gastric mucosa from ethanol induced injury by enhancing the antioxidant defense system. The protective effects of BCT were confirmed by gross pathological and histopathological examinations of stomach tissues. Ethanol-induced gastric lesions are characterized by hemorrhage, erosion, ulceration, and hyperemia. In our study, we observed gastric mucosal changes in animals treated with absolute ethanol, whereas the extent of the changes was reduced in animals treated with BCT and ethanol compared with animals treated with ethanol alone. Based on these observations, oral administration of BCT appears to attenuate ethanol-induced acute gastric injury.

In addition, we evaluated the safety of BCT in an acute toxicity study. BCT did not cause any adverse effects in animals at up to 5000 mg/kg. The information obtained from the acute toxicity study is useful for determining the safety of the test material to protect human health. Thus, our results indicate that BCT may be a very safe material.

## Conclusions

BCT reduced the histopathological changes in stomach tissue induced by ethanol intake due to decreased lipid peroxidation and enhancement of the antioxidant defense system. These findings indicate that BCT can protect against ethanol induced gastric injury by enhancing the antioxidant defense system. We suggest that BCT may be a useful material for treating acute gastric injury.

## Competing interest

There are no competing financial interests.

## Authors’ contributions

ISS, HKS and MYL participated in the design of the study data analyses and manuscript preparation. WYJ conducted the biochemical analyses for gastric tissue and SWC evaluated the safety of BCT throughout acute toxicity study. All authors read and approved the final manuscript.

## Pre-publication history

The pre-publication history for this paper can be accessed here:

http://www.biomedcentral.com/1472-6882/13/170/prepub

## References

[B1] Hamaishi K Kojima R Ito M Anti-ulcer effect of tea catechin in rats Bio Pharm Bull 2006 29 2206 2213 10.1248/bpb.29.2206 17077516

[B2] Rao CV Vijayakumar M Protective effect of (+)-catechin against gastric mucosal injury induced by ischaemia-reperfusion in rats J Pharm Pharmacol 2007 59 1103 1107 10.1211/jpp.59.8.0007 17725852

[B3] Sener-Muratoglu GS Paskaloglu K Arbak S Hurdag C Ayanoglu-Dulger G Protective effects of famotidine, omeprazole, and melatonin against acetylsalicylic acid-induced gastric damage in rats Dig Dis Sci 2001 46 318 30 10.1023/A:1005652815921 11281181

[B4] Silva MI Moura BA Neto MR Tome Ada R Rocha NF de Carvalho AM Macedo DS Vasconcelos SM de Sousa DP Viana GS de Sousa FC Gastroprotective activity of isopulegol on experimentally induced gastric lesions in mice: investigation of possible mechanism of action Naunyn Schmiedebergs Arch Pharmacol 2009 380 233 245 10.1007/s00210-009-0429-5 19479241

[B5] Liu CF Lin CC Lin MH Lin YS Lin SC Cytoprotection by propolis ethanol extract of acute absolute ethanol-induced gastric mucosal lesions Am J Chin Med 2002 30 245 254 10.1142/S0192415X02000387 12230013

[B6] Yam MF Ang LF Salman M Ameer OZ Lim V Ong LM Ahmad M Asmawil MZ Basir R Orthosiphon stamineus leaf extract protects against ethanol-induced gastropathy in rats J Med Food 2009 12 1089 1097 10.1089/jmf.2008.0005 19857074

[B7] Jaiswal SK Rao CV Sharma B Mishra P Das S Dubey MK Gastroprotective effect of standardized leaf extract from Argyreia speciosa on experimental gastric ulcers in rats J Ethnopharmacol 2011 137 341 344 10.1016/j.jep.2011.05.028 21658440

[B8] Ibrahim IA Qader SW Abdulla MA Nimir AR Abdelwahab SI Al-Bayaty FH Effects of Pithecellobium jiringa ethanol extract against ethanol-induced gastric mucosal injuries in Sprague–Dawley rats Molecules 2012 17 2796 2811 10.3390/molecules17032796 22395408 PMC6268751

[B9] Shin HM Morgan KG Vasodilation by banhabackchulchunmatang, a Chinese medicine, is associated with negative modulation of PKCα activation and NO production Life Sci 2003 74 723 732 10.1016/j.lfs.2003.06.040 14654165

[B10] Maclean W Lyttleton J Phelgm Damp. Clinical handbook of internal medicine 2002 2 Campbeltown, Australia: University of Western Sydney Macarthur 548 551

[B11] Lee HJ Seong YJ Moon KJ Kim JB Kim GW Shin HM Enhanced vasorelaxation of banhabackchulchunma-tang and involved mechanism Kor J Oreintal Physiol Pathol 2005 19 1311 1316

[B12] Sakurai T Sugawara H Saito K Kano Y Effects of the acetylene compound from Atractylodes rhizome on experimental gastric ulcers induced by active oxygen species Biol Pharm Bull 1994 17 1364 1368 10.1248/bpb.17.1364 7874060

[B13] Lee YS Han OK Park CW Yang CH Jeon TW Yoo WK Kim SH Kim HJ Pro-inflammatory cytokine gene expression and nitric oxide regulation of aqueous extracted Astragali radix in RAW 264.7 macropharge cells J Ethnopharmacol 2005 100 289 294 10.1016/j.jep.2005.03.009 15871914

[B14] Li CQ He LC Jin JQ Atractylenolide I and atractylenolide III inhibit lipopolysaccharide-induced TNF-alpha and NO production in macrophages Phytoher Res 2007 21 347 353 10.1002/ptr.2040 17221938

[B15] Rhyu DY Kang KS Sekiya M Yokozawa T Antioxidant effect of Wen-Pi-Tang and its component crude drugs on oxidative stress Am J Chin Med 2007 35 127 137 10.1142/S0192415X07004680 17265557

[B16] Yeo M Kim DK Cho SW Hong HD Ginseng, the root of Panax ginseng C.A. Meyer, protects enthanol-induced gastric damages in rat through the induction of cytoprotective heat-shock protein 27 Dig Dis Sci 2008 53 606 613 10.1007/s10620-007-9946-6 17763949

[B17] Hwang SM Lee YJ Kang DG Lee HS Anti-inflammatory effect of Gastrodia elata rhizome in human umbilical vein endothelial cells Am J Chin Med 2009 37 395 406 10.1142/S0192415X09006916 19507281

[B18] Kim YO Kim HJ Kim GS Park HG Lim SJ Seong NS Ham YW Lee SD Jang KH Jung KH Chung JH Kang SA Panax ginseng protects against global ischemia injury in rat hippocampus J Med Food 2009 12 71 76 10.1089/jmf.2007.0614 19298198

[B19] Rios JL Chemical constituents and pharmacological properties of Poria cocos Planta Med 2011 77 681 691 10.1055/s-0030-1270823 21347995

[B20] Robert A Nezamis JE Lancaster C Hanchar AJ Cytoprotection by prostaglandins in rats. Prevention of gastric necrosis produced by alcohol, HCl, NaOH, hypertonic NaCl, and thermal injury Gastroenterol 1979 77 433 443 456839

[B21] Lee MY Shin IS Jeon WY Seo CS Ha H Huh JI Shin HK Protective effect of Bojungikki-tang, a traditional herbal formula, against alcohol-induced gastric injury in rats J Ethnopharmacol 2012 142 346 353 10.1016/j.jep.2012.04.043 22580157

[B22] Bilici D Suleyman H Banoglu ZN Kiziltunc A Avci B Ciftcioglu A Bilici S Melatonin prevents ethanol-induced gastric mucosal damage possibly due to its antioxidant effect Dig Dis Sci 2002 47 856 861 10.1023/A:1014764705864 11991621

[B23] Jainu M Devi CS Gastroprotective action of *Cissus quadrangularis* extract against NSAID induced gastric ulcer: role of proinflammatory cytokines and oxidative stress Chem Biol Interact 2006 161 262 270 10.1016/j.cbi.2006.04.011 16797507

[B24] Cadirci E Suleyman H Aksoy H Halici Z Ozgen U Koc A Ozturk N Effects of Onosma armeniacum root extract on ethanol-induced oxidative stress in stomach tissue of rats Chem Biol Interact 2007 170 40 48 10.1016/j.cbi.2007.06.040 17681286

[B25] Halliwell B Aeschbach R Loliger J Aruoma OI The characterization of antioxidants Food Chem Toxicol 1995 33 601 617 10.1016/0278-6915(95)00024-V 7628797

[B26] Zamora Rodriguez ZB Conzalez Alvarez R Guanche D Merino N Hemandez Rosales F Menendez Cepero S Alonso Gonzalez Y Schulz S Antioxidant mechanism in involved in the gastroprotective effects of ozonized sunflower oil in ethanol-induced ulcers in rats Mediators Inflamm 2007 200 7 65873 17497036 10.1155/2007/65873 PMC1804299

[B27] Oyagi A Ogawa K Kakino M Hara H Protective effects of a gastrointestinal agent containing Korean red ginseng on gastric ulcer models in mice BMC Complement Altern Med 2010 10 45 10.1186/1472-6882-10-45 20718962 PMC2936409

[B28] Vasanthkumar M Parameswari RP Vijaya Kumar V Sangeetha MK Gayathri V Balaji Raghvendran H Chamundeeswari D Vasanthi HR Anti-ulcer role of herbomineral Siddha drug – Thamira parpam on experimentally induced gastric mucosal damage in rats Hum Exp Toxicol 2010 29 161 173 10.1177/0960327109357217 20051456

[B29] Lemos LM Martins TB Tanajura GH Gazoni VF Bonaldo J Strada CL Silva MG Dall’oglio EL de Sousa Junior PT Martins DT Evaluation of antiulcer activity of chrmanone fraction from Calophyllum brasiliesnse Camb J Ethnopharmacol 2012 141 432 439 10.1016/j.jep.2012.03.006 22425905

[B30] Devi RS Narayan S Vani G Shyamala Devi CS Gastroprotective effect of Terminalia arjuna bark on diclofenac sodium induced gastric ulcer Chem Biol Interact 2007 167 71 83 10.1016/j.cbi.2007.01.011 17327128

[B31] Chelikani P Fita I Loewen PC Diversity of structures and properties among catalases Cell Mol Life Sci 2004 61 192 208 10.1007/s00018-003-3206-5 14745498 PMC11138816

[B32] Farias-Silva E Cola M Calvo TR Barbastefano V Ferreira AL De Paula Michelatto D de Almeida AC A Hiruma-Lima CA Vilegas W Brito AR Antioxidant activity of indigo and its preventive effect against ethanol-induced DNA damage in rat gastric mucosa Planta Med 2007 73 1241 1246 10.1055/s-2007-981613 17973201

